# Childhood Speech Impairment and Dementia Risks Among U.S. Older Adults

**DOI:** 10.1111/1460-6984.70274

**Published:** 2026-06-13

**Authors:** Haowei Wang, Shu Xu, Yalian Pei

**Affiliations:** ^1^ Department of Sociology and Aging Studies Institute, Maxwell School of Citizenship and Public Affairs Syracuse University Syracuse New York USA; ^2^ Survey Research Center, Institute for Social Research University of Michigan Ann Arbor Michigan USA; ^3^ Department of Communication Sciences and Disorders, The College of Arts and Sciences Syracuse University Syracuse New York USA

**Keywords:** cumulative advantage/disadvantage, dementia, life‐course, speech impairment

## Abstract

**Background:**

Speech problems in childhood have profound implications for learning, communication, as well as the development of social and cognitive skills in adulthood. However, research has yet examined how early life speech problems may be associated with subsequent dementia risks in later life.

**Aims:**

Using nationally representative data from the longitudinal Health and Retirement Study, this study aimed to investigate how the experience of speech problems before age 16 was associated with the risk of dementia among older adults aged 50 and older.

**Methods and Procedures:**

The analysis pooled the full life history information about childhood speech problems for *N* = 17,863 participants who had one to eleven observations for cognitive and sociodemographic information from 2000 to 2020. We constructed person‐interval data and estimated discrete‐time event history models to examine risks for developing dementia, which was measured using a validated Langa‐Weir classification of cognitive function.

**Results:**

Results showed that older adults with childhood speech problems had a higher risk of developing dementia in later life, compared to those who never had speech problems in childhood. The effect of childhood speech problems on cognitive function was partially explained by economic status and health conditions in midlife.

**Conclusions and Implications:**

Findings highlight the long‐arm implications of early life health adversity and suggest needs for screening and early intervention programs for childhood speech impairment.

**WHAT THIS PAPER ADDS:**

*What is already known on this subject*

- Literature had documented various developmental and health outcomes of childhood speech impairment. This research addresses the gap in the literature by examining the long‐term implications of childhood speech impairment on dementia risk in later life.

*What this paper adds to existing knowledge*

- Using longitudinal data from the *Health and Retirement Study*, this study investigated how the experience of childhood speech impairment was associated with the risk of dementia among U.S. older adults. We found about 3% of U.S. older adults had speech impairments in childhood, and they had a higher risk of developing dementia in later life compared to those who did not have speech impairments in childhood.

*What are the potential or actual clinical implications of this work?*

- Findings suggest needs for screening and early intervention programs for childhood speech impairment.

## 1 Introduction

Speech impairment refers to diminished abilities to produce speech sounds, negatively impacting the clarity, fluency, and overall effectiveness of spoken communication. This condition commonly arise from speech sound disorders and fluency disorders (American Speech‐Language‐Hearing Association 1993). About 5% of children in the United States aged 3 to 17 experience a speech disorder lasting a week or longer within a 12‐month period (Black et al. 2015). Among young children, the prevalence of speech sound disorders is estimated to be 8%–9% (Law et al. 2000, Shriberg et al. 1999), with prevalence in boys being 1.5–1.8 times than higher in girls (Wren et al. 2016). Stuttering, affecting 1% of the U.S. population, is most common in children aged 2 to 6 and occurs two to three times more frequently in boys than in girls. While many children may outgrow stuttering, approximately 25% may experience persistent developmental stuttering that continues into adulthood (Boyle et al. 2010, Yairi and Ambrose 2013). Still, speech impairment is often underdiagnosed and overlooked as a disability in childhood (Halfon et al. 2012, Prizant et al. 1990).

Like sensory impairments and other childhood stressors, early life speech impairment is likely to increase the risk of dementia due to cumulative disadvantages over time (Hu et al. 2024, Nilaweera et al. 2022). Speech and language development is a crucial aspect of cognitive development and impaired speech or delayed language acquisition can hinder cognitive development in various domains, such as attention, memory, perception, and problem‐solving (Alloway and Archibald 2008, Liao et al. 2015), further affecting the acquisition of social and academic skills. Cognitive development throughout life, particularly in early years, significantly shape an individual's susceptibility to cognitive impairment and dementia in later life (Huh et al. 2024). For instance, children with speech sound disorders often exhibit weaknesses in processing phonological representations, which may hinder their literacy development and lead to reading difficulties (Anthony et al. 2011, Lewis et al. 2019). Longitudinal studies consistently show that, compared to those without speech impairments, those with speech impairment perform poorer in areas such as communication, cognitive and academic development, educational attainment, and occupational status (Johnson et al. 1999, 2010). Reduced educational attainment and occupational status (Conti‐Ramsden et al. 2013, Johnson et al. 2010), frequently observed in individuals with early speech impairments, are associated with lower cognitive reserve—a protective factor against dementia. Cognitive reserve is built through engaging in complex mental activities, often expressed as having higher education levels, occupational complexity, and mentally stimulating lifestyle pursuits. These activities help strengthen neural connections and may delay the onset of dementia symptoms (Fratiglioni and Wang 2007, Valenzuela and Sachdev 2006). In contrast, early‐life speech impairment may contribute to long‐term cognitive vulnerability and place individuals at a disadvantage for building resilience against cognitive decline.

Moreover, speech impairments can lead to challenges in forming and maintaining social relationships (McCormack et al. 2009), which are vital for psychological well‐being and cognitive health. Social networks provide access to social interactions, emotional support, and resources. Additionally, speech impairment is recognized as a risk factor for anxiety and depression (Beitchman et al. 2001, Lee et al. 2020, Lim and Lum 2024). The negative social and emotional impact of speech impairments are evident as early as school age, where affected children often face peer rejection and social isolation (Conti‐Ramsden et al. 2013, Hitchcock et al. 2015), as well as issues with interpersonal trust (Clarke et al. 2023). The challenges may persist into adulthood, even when accounting for factors such as age, gender, and overall health (Palmer et al. 2016). Over time, this accumulation of social and emotional disadvantages may create a compounding effect, further increasing vulnerability to dementia in later life. However, there is still no empirical research examining the impacts of childhood speech impairment on later life cognitive function.

Using data from a nationally representative longitudinal survey, the purpose of this study is to investigate the association between childhood speech impairment and cognitive health among U.S. older adults. We also examine midlife biopsychological pathways linking the association between childhood speech impairment and dementia risks. Midlife socioeconomic status (SES) may operationalize the cognitive reserve pathway, as speech impairment constrains educational and occupational attainment, reducing lifelong access to cognitively stimulating environments that buffer against neurodegeneration (Stern 2012). Midlife health conditions operationalize the chronic stress pathway: chronic psychosocial adversity associated with early speech difficulties, including peer victimization and academic frustration, may elevate cumulative cardiometabolic burden, accelerating cerebrovascular damage over decades (Lupien et al. 2009; Livingston et al. 2020). Social isolation, while not directly measured, is partially captured through midlife SES pathway given the close link between speech impairment, social marginalization, and occupational exclusion (Johnson et al. 2010, McCormack et al. 2009). We will examine whether and to what extend these midlife risk factors can explain the relationship between childhood speech impairment and later life dementia risk.

## 2 Materials and Methods

### 2.1 Data and Sample

Data was drawn from the longitudinal *Health and Retirement Study* (HRS), which is a nationally representative dataset of Americans aged 50 years and older. The HRS used a multi‐stage area probability sample in survey design and over sampled Blacks, Hispanics, and Floridians (Servais 2010). The survey has been conducted every two years since 1992 by the University of Michigan and is funded by the National Institute on Aging (NIA U01AG009740). The initial sample included respondents who were born 1931–1941 and their spouses/partners (the HRS cohort), and the HRS added new cohorts of War Baby Cohorts in 1998 (WB, born 1942–1947), the Early Baby Boomer Cohort in 2004 (EBB, born 1948–1953), the Mid Baby Boomer cohort in 2010 (MBB, born 1954–1959), and the Late Baby Boomer cohort in 2016 (LBB, born 1960–1965). The HRS contained a rich array of information on physical and mental health status, health behaviors, retirement planning, income, and family structure.

HRS retrospectively collected life history information about early life events, including health and impairments in childhood; cognitive function was measured repeatedly since 2000. Thus, the sample consists of community dwelling respondents aged 50 and older in the survey and had nonmissing measures for childhood speech impairment and cognition. Participants with cognitive impairment at baseline were excluded prior to all analyses, so retrospective childhood recall was obtained exclusively from cognitively intact respondents. The final analytical sample included *N* = 17,863 participants with 119,674 observations from 2000–2020, and 619 respondents of them have self‐identified speech impairment in childhood.

### 2.2 Measures

*Childhood speech impairment* was measured by life history information about respondents. Starting from 2008, respondents were retrospectively asked whether they had speech problems before age 16 (yes or no). HRS also fielded Life History Mail Surveys (LHMS) from 2015 to 2019 fall, specifically collecting information about childhood health, job history, and marital transition over the life course. By combining HRS longitudinal data and LHMS data, we created a binary measure indicating whether the respondent ever had speech impairment in childhood.

*Dementia* was assessed by the validated Langa‐Weir classification of cognitive function (Langa et al. 2023). HRS includes a set of questions to measure the cognitive function of respondents, including (1) immediate and delayed recall (score 0–20), (2) calculating serial 7's (score 0–5), and (3) backward counting (score 0–2; in total score 0 to 27). For respondents who had proxies (about 10% of HRS respondents) to assess their cognitive function, they were rated based on instrumental activities of daily living (ADL; score 0–5), proxy assessment of respondent's memory (score 0–4), and interviewer's assessment of respondent's cognition (score 0–2; in total score 0–11). The Langa‐Weir classification considers respondents as having dementia if they scored 6 or lower on the self‐reported measure or proxy reports. This classification has been validated and widely used in assessing dementia using HRS (Langa et al. 2017, Umberson et al. 2020).

Midlife economic status was measured by annual household income (transformed by natural log) and household wealth (in quartiles) from the baseline survey when respondents first entered the HRS. Total household income only included the respondent and spouse, excluding other household members (Servais 2010). Midlife health behaviors included currently smoking, currently drinking alcohol, and Body Mass Index (BMI; respondents with top and bottom 5% BMI were excluded). Moreover, we assessed midlife health conditions including any limitations with ADL, including dressing, eating, bathing, getting in and out of bed, and walking across a room), a count number of four cardiometabolic conditions (i.e., heart disease, stroke, diabetes, and hypertension), and a count number of eight depressive symptoms (Radloff 1977).

The analysis also considered respondents’ demographic characteristics, including age (at baseline), gender (1 = female and 0 = male), race and ethnicity (i.e., non‐Hispanic White, non‐Hispanic Black, Hispanic, and non‐Hispanic other groups), marital status (1 = married, 0 = not married), and education (in years). Time‐invariant control variables were taken from the HRS tracker file and FAT files, and time‐varying control variables were used from the RAND Longitudinal File 2000 (v2).

### 2.3 Analytic Strategy

First, we stratified the sample by whether respondents had speech impairment before at 16 and compared the differences by key demographic, socioeconomic, and midlife health behaviors and health conditions. Estimates were adjusted using sample weights from the HRS to be representative of U.S. older adults.

Next, we estimated a series of discrete‐time event history models to examine the risk of dementia from 2000 to 2020. We created person‐interval data for respondents who were dementia free at baseline, with each respondent could have contributed to one to eleven 2‐year intervals. At each following observation since baseline, respondents could be censored by developing dementia, dying, or dropping out of the survey. We estimated multinomial logistic regression models to predict dementia incidence (with dying or dropping out combined as the competing risk). The base model included childhood speech impairment and key demographic variables (i.e., baseline age, gender, race and ethnicity, marital status, and education). We then added midlife economic conditions (i.e., income and wealth), midlife health behaviors (i.e., smoking, drinking alcohol, and BMI), and midlife health conditions (i.e., any ADL limitation, cardiometabolic symptoms, and depressive symptoms) to the base model to predict dementia incidence.

In order to examine the pathways, we used the Karlson‐Holm‐Breen (KHB) method (Breen et al. 2021) to illustrate the indirect effects of economic conditions, health behaviors, and health conditions at midlife in the association between childhood speech impairments and dementia incidence. For the statistically significant pathways identified in the KHB results, we conducted regression analyses to examine how childhood speech impairment was associated with different midlife health risks.

The vast majority of respondents had complete information on all analytic variables after applying the exclusion criteria to create the analytical samples. Missing values were handled by listwise deletion and analyzed using Stata/MP Version 18.

## 3 Results

Descriptive characteristics of sample participants by childhood speech impairment history are presented in Table 1. An estimated 3.11% of U.S. adults aged 50 and older experienced speech impairment before age 16. Among them, 11.88% developed dementia during 2000 to 2020. Older adults with childhood speech impairment were, on average, younger (55.38 vs. 57.98), less likely to be female (40.47% vs. 51.79%), more likely to be non‐Hispanic Black (13.81% vs. 10.29%) or non‐Hispanic other race groups (7.71% vs. 4.93%), and had more years of education (13.59 vs. 13.24) compared to their counterparts without childhood speech impairment. Although there revealed no statistical difference in income and wealth, we found that older adults with childhood speech impairment had a higher BMI (29.31 vs. 28.04) and more depressive symptoms (1.83 vs. 1.45), and were more likely to have ADL limitations (32.14% vs. 20.39%) in relative to those who did not have speech impairment in childhood based on bivariate analyses.

**TABLE 1:** Descriptive characteristics of HRS respondents by childhood speech impairment.

	Had speech impairment before age 16 (*n* = 619)		No speech impairment before age 16 (*n* = 17,244)		Diff.
Variables	*M*/*%*	(*SD*)	*M/%*	(*SD*)	
Childhood speech Impairment	3.11%				
Dementia incidence (baseline to 2020)	11.88%		11.09%		
*Demographic characteristics*					
Age (baseline)	55.38	(5.31)	57.98	(7.85)	^***^
Female	40.47%		51.79%		^***^
Race and ethnicity					^*^
Non‐Hispanic White	72.78%		76.11%		
Non‐Hispanic Black	13.81%		10.29%		
Non‐Hispanic Other	7.71%		4.93%		
Hispanic	5.69%		8.66%		
Married	64.83%		67.30%		
Education (in years)	13.59	(2.54)	13.24	(2.95)	^**^
*Economic status (midlife baseline)*					
Household income ($)	101,961.2		85,657.5		
Household wealth ($)	385,571.1		404,336.1		
*Health behaviors (midlife baseline)*					
Currently smoking	19.18%		19.73%		
Currently drinking alcohol	45.27%		43.11%		
Body Mass Index	29.31	(4.98)	28.04	(5.23)	^***^
*Health conditions (midlife baseline)*					
Cardiometabolic conditions^b^	0.71	(0.83)	0.64	(0.81)	
Any ADL disability^c^	32.14%		20.39%		^**^
Depressive symptoms^d^	1.83	(2.07)	1.45	(1.95)	^**^

*Notes*: HRS, Health and Retirement Study.

Respondent *N* = 17,863; Observation *N* = 119,674.

^a^ Rated from 1 = excellent to 5 = poor

^b^ Count number of four cardiometabolic conditions including heart disease, stroke, diabetes, and hypertension.

^c^ Any disability reported for five activities of daily living (ADL), including dressing, eating, bathing, getting in and out of bed, and walking across a room.

^d^ Count number of eight depressive symptoms.

Statistics are weighted using the HRS sample weights at baseline.

* *p* < 0.05. ^**^ *p* < 0.01. ^***^ *p* < 0.001.

Table 2 presents results from discrete‐time hazard models for the association of childhood speech impairment and dementia risks among older adults. Model 1 suggested that experiencing speech impairment in childhood was significantly associated with a higher risk of dementia incidence (RRR = 1.63, *p* < 0.001). The association remained significant with the inclusion of midlife economic status (Model 2: RRR = 1.58, *p* < 0.001) and midlife health behaviors (Model 3: RRR = 1.57, *p* < 0.001). Lastly, we added midlife health conditions to the previous model and the association between childhood speech impairment and dementia incidence risk were reduced but remained significant (Model 4: RRR = 1.45, *p* < 0.01). Moreover, results from Model 4 revealed that lower income and wealth, smoking, more cardiometabolic symptoms, ADL limitations, and a greater number of depressive symptoms were associated with higher risks of dementia incidence among older adults.

**TABLE 2:** Multinomial logistic regression results for associations between childhood speech impairment and dementia risks.

	Dementia vs. No dementia							
	M1		M2: M1+ Economic conditions		M3: M2+ Health behavior		M4: M3+ Health conditions	
	RRR	(*SE*)	RRR	(*SE*)	RRR	(*SE*)	RRR	(*SE*)
Childhood speech impairment	1.63^***^	(0.21)	1.58^***^	(0.21)	1.57^***^	(0.21)	1.45^**^	(0.21)
*Demographic characteristics*								
Age (baseline)	1.09^***^	(0.00)	1.09^***^	(0.00)	1.09^***^	(0.00)	1.09^***^	(0.00)
Female	0.83^***^	(0.04)	0.82^***^	(0.04)	0.81^***^	(0.04)	0.87^*^	(0.05)
Race and ethnicity								
Non‐Hispanic White (reference)	—	—	—	—	—	—	—	—
Non‐Hispanic Black	2.33^***^	(0.14)	2.12^***^	(0.13)	2.08^***^	(0.14)	1.97^***^	(0.13)
Non‐Hispanic Other	1.78^***^	(0.29)	1.69^**^	(0.27)	1.59^**^	(0.26)	1.45^*^	(0.25)
Hispanic	1.52^*^**	(0.12)	1.38^***^	(0.11)	1.43^***^	(0.12)	1.56^***^	(0.14)
Married	0.48^***^	(0.02)	0.52^***^	(0.03)	0.52^***^	(0.03)	0.52^***^	(0.03)
Education (in years)	0.86^***^	(0.01)	0.88^***^	(0.01)	0.88^***^	(0.01)	0.89^***^	(0.01)
Household income (logged)	—	—	0.94^***^	(0.02)	0.94^***^	(0.02)	0.96^**^	(0.02)
Household wealth (in quartiles)								
1st quartile (reference)	—	—	—	—	—	—	—	—
2nd quartile	—	—	0.81^***^	(0.05)	0.82^**^	(0.05)	0.82^**^	(0.05)
3rd quartile	—	—	0.76^***^	(0.05)	0.78^***^	(0.06)	0.81^**^	(0.06)
4th quartile	—	—	0.77^***^	(0.06)	0.82^*^	(0.06)	0.85^*^	(0.07)
*Health behaviors*								
Currently smoking	—	—	—	—	1.39^***^	(0.09)	1.37^***^	(0.09)
Currently drinking alcohol	—	—	—	—	0.85^**^	(0.05)	0.91	(0.05)
Body Mass Index	—	—	—	—	1.00	(0.00)	0.99^**^	(0.01)
*Health conditions*								
Cardiometabolic conditions^a^	—	—	—	—	—	—	1.21^***^	(0.04)
Any ADL disability^b^	—	—	—	—	—	—	1.10^**^	(0.03)
Depressive symptoms^c^	—	—	—	—	—	—	1.06^***^	(0.01)

*Notes*: RRR = Relative Risk Ratio.

^a^ Count number of four cardiometabolic conditions including heart disease, stroke, diabetes, and hypertension.

^b^ Any disability reported for five activities of daily living (ADL), including dressing, eating, bathing, getting in and out of bed, and walking across a room.

^c^ Count number of eight depressive symptoms.

^*^ *p* < 0.05. **p < 0.01. ***p < 0.001.

Table 3 summarizes the KHB results decomposing the direct and indirect effects of childhood speech impairment on risks of dementia incidence through different midlife pathways. Results showed that economic status and health conditions, rather than health behaviors, partially explained the association of childhood speech impairment with later life dementia incidence. We then tested for indirect effect for each specific pathway and found wealth (1.74%), cardiometabolic symptoms (1.84%), ADL limitations (2.31%), and depressive symptoms (4.94%) explained different proportions of the total effect, respectively. To further illustrate the associations between childhood speech impairment and midlife health risks, we conducted regression analyses, and results were presented in Table 4, showing that childhood speech impairment was significantly associated with lower wealth (OR = 0.84, *p* < 0.001), more cardiometabolic symptoms (B = 0.84, *p* < 0.001), higher risks of ADL disability (OR = 1.65, *p* < 0.01), and more depressive symptoms (B = 1.49, *p* < 0.001).

**TABLE 3:** KHB results for pathways liking childhood speech impairment to dementia risks.

	Midlife pathways		
	Economic conditions^a^	Health behavior^b^	Health conditions^c^
Total effect	0.47	0.47	0.42
Direct effect	0.45	0.47	0.35
% explained by indirect effects	1.79%	0	6.80%

*Notes*: KHB = Karlson‐Holm‐Breen method.

^a^ Include household income and wealth.

^b^ Include currently smoking, currently drinking alcohol, and body mass index (BMI).

^c^ Include a count number of cardiometabolic symptoms, any activity of daily living (ADL) disability, and a count number of depressive symptoms.

**TABLE 4:** Selected regression results for associations between childhood speech impairment and midlife health risks.

	Model 1: Wealth		Model 2: Cardiometabolic symptoms		Model 3: Any ADL disability		Model 4: Depressive symptoms	
	OR	(*SE*)	B	(*SE*)	OR	(*SE*)	B	(*SE*)
Childhood speech impairment	0.84^***^	(0.03)	1.10^***^	(0.01)	1.66^***^	(0.08)	1.49^***^	(0.04)
*Demographic characteristics*								
Age (baseline)	1.05^***^	(0.00)	1.01^***^	(0.00)	1.00	(0.00)	0.98^***^	(0.00)
Female	1.03^**^	(0.01)	0.89^***^	(0.00)	1.36^***^	(0.03)	1.27^***^	(0.01)
Race and ethnicity								
Non‐Hispanic White (reference)	—	—	—	—	—	—	—	—
Non‐Hispanic Black	0.23^***^	(0.00)	1.38^***^	(0.01)	1.77^***^	(0.04)	1.38^***^	(0.02)
Non‐Hispanic Other	0.49^***^	(0.02)	1.15^***^	(0.02)	1.46^***^	(0.08)	1.59^***^	(0.05)
Hispanic	0.40^***^	(0.01)	0.98^*^	(0.01)	1.32^***^	(0.04)	1.46^***^	(0.03)
Married	2.79^***^	(0.03)	0.98^***^	(0.00)	0.65^***^	(0.01)	0.55^***^	(0.01)
Education (in years)	1.26^***^	(0.00)	0.98^***^	(0.00)	0.89^***^	(0.00)	0.90^***^	(0.00)
% total effect explained	1.74%		1.85%		2.31%		4.94%	

*Notes*: Model 1 is based on ordered logit regression model; Model 2 and Model 4 are based on linear regression models; Model 3 is based on logit regression model.

OR = odds ratio.

Wealth: transformed into quartiles. Cardiometabolic symptoms: a count number of four conditions including heart disease, stroke, diabetes, and hypertension. ADL: any disability reported for five activities of daily living, including dressing, eating, bathing, getting in and out of bed, and walking across a room. Depressive symptoms: a count number of eight depressive symptoms.

^*^ *p* < 0.05. **p < 0.01. ***p < 0.001.

We conducted the following sensitivity analyses to check the robustness of analytical results. First, we estimated models for respondents who aged 65 and older at the baseline survey because they were at a higher risk of dementia incidence due to their age. Second, we conducted analyses only for non‐proxy respondents to test whether the results were different by participation modes. Third, we re‐estimated the primary multinomial logistic regression including respondents with dementia at baseline; we also estimated a survey‐weighted logistic regression with baseline dementia status as the outcome, finding that childhood speech impairment was significantly associated with early‐onset dementia. Fourth, to address concerns regarding shared etiology, survival bias, and residual confounding, we added three childhood‐ascertained covariates to the models: childhood family SES, childhood self‐rated health, and other childhood adversities (family financial difficulty and father unemployment). Effect estimates were not materially attenuated following these additions. To maintain parsimony in the primary models and preserve focus on childhood speech impairment as the key exposure, these additional childhood covariates were included as sensitivity analyses rather than in the main specification. Fifth, we recoded educational attainment into five categories (less than high school, GED, high school graduate, some college, and college or above), collapsed into three strata for education‐stratified models and a multiplicative interaction test (childhood speech impairment × education). The speech–dementia association remained statistically significant across all strata, and the interaction term was not statistically significant. Across all five specifications, findings were consistent with the primary analysis, supporting the robustness of the observed association between childhood speech impairment and late‐life dementia incidence.

## 4 Discussion

Taking a lifespan perspective, this study adds to the literature on how early life health adversity such as speech impairment can have a long‐lasting implication on later‐life cognitive health. Existing literature recognizes that childhood speech impairment may be associated with personal development and wellbeing, including developmental tasks (Gaines and Missiuna 2007, Schuele 2004), psychological well‐being (Beitchman et al. 2014, Lim and Lum 2024), and social relationships in adulthood (Palmer et al. 2016). Children with speech or language impairment may have persistent language problems in adolescence and adulthood, and they also have elevated risks of developmental disorders (e.g., intellectual disability and autism spectrum disorder) (Ek et al. 2012, Jansen et al. 2020). Due to problems with communication skills, individuals with childhood speech impairment are less likely to stay in education or employment (Conti‐Ramsden and Durkin 2016, Johnson et al. 2010), and they are also at higher risks of psychiatric disorders (Beitchman et al. 2014, Lim and Lum 2024) and substance and alcohol misuse (Armstrong et al. 2017). Although developmental disorders, psychological problems, and lack of social support are well established risk factors for dementia, there is still no study connecting childhood speech impairment to later life dementia. Our findings provide evidence that older adults with childhood speech impairment have greater risks of developing dementia compared to peers without the condition. This finding is based on a longitudinal observation of from 2000 to 2020 after accounting for sociodemographic characteristics and biopsychosocial pathways in midlife.

Unlike other disabilities or sensory impairments, speech impairment is often overlooked in children due to various reasons (McGregor 2020, Prelock et al. 2008). Parents may not recognize the symptoms of speech impairment and sometimes attribute the symptoms as shyness, introversion, or a phase that the child will outgrow. Pediatricians may include language speech impairment as part of screenings, but the implementation, procedures, and following services are not sufficient (Hendricks et al. 2019). Additionally, speech impairments can co‐occur with other developmental or learning disorders, such as autism (Schaeffer et al. 2023), ADHD (Sciberras et al. 2014), or hearing loss (Blamey et al. 2001). In such cases, the speech issue may be overshadowed by the more prominent condition. Using longitudinal data from HRS, we found over 3% of US older adults have experienced speech impairment in childhood and these older adults had higher risks for dementia incidence in later life. Childhood speech impairment was ascertained via a single retrospective self‐report item. Although participants with baseline cognitive impairment were excluded, systematic differences in recall propensity cannot be fully excluded. To the extent that resulting misclassification is non‐differential with respect to prospectively ascertained dementia status, effect estimates are likely biased toward the null, meaning the true association may be stronger than reported.

This study also investigated a number of midlife risk factors that may be associated with childhood speech impairment and potentially increase the risk of dementia in later life. We found that childhood speech impairment was associated with economic conditions (i.e., lower level of household wealth) and physical health conditions (i.e., more cardiometabolic symptoms, higher risks of ADL disability, and more depressive symptoms) when respondents first entered the survey at age 50. Further, our results showed that economic status and physical health conditions were associated with dementia risks in later life. These findings reinforce the notion that childhood speech impairment may be a marker of broader developmental vulnerabilities that persist across the life course. Socioeconomic disadvantage in midlife may reflect cumulative disadvantage over time for individuals with a history of childhood speech impairment, including lower educational attainment, fewer occupational opportunities, and reduced access to healthcare—factors that can limit cognitive reserve and accelerate cognitive aging. The relationship between childhood speech impairment and dementia risks were attenuated after controlling for these midlife dementia risks factors—although remaining statistically significant, suggesting the modeled risk factors can only partially explain the pathways linking childhood speech impairment to dementia incidence. Future research should explore other potential pathways, such as chronic stress, social isolation, and lifelong cognitive engagement, which may also contribute to cognitive decline in this population. Contrary to our hypothesis about the role of unhealthy behaviors, there was no significant difference in smoking and drinking alcohol between older adults with and without childhood speech impairment. Future research could explore the interaction effects between health behaviors, health conditions, and dementia risk, as well as examine other indicators of health behaviors, such as the frequency and amount of smoking and drinking. Although older adults with childhood speech impairment had a higher level of BMI, the indirect effect of BMI was not statistically significant. Findings on the association between BMI and dementia risk have been heterogenous. For example, the relationship between BMI and dementia risk may vary across age groups, and the risk is influenced not only by the absolute BMI value but also by changes in BMI patterns over time ^49^.

Several sources of survival and selection bias warrant consideration when interpreting the present findings. Regarding survival bias, HRS‐based research examining early‐life exposures is subject to left‐truncation bias from selective survival to enrollment at approximately age 50. If childhood speech impairment is associated with elevated pre‐enrollment mortality, individuals with more severe impairment are systematically underrepresented, producing a survivor cohort that is healthier than the underlying population. This biases effect estimates toward the null, meaning the reported associations likely underestimate the true population‐level relationship. Sensitivity analysis adjustment for childhood self‐rated health partially addresses the health composition of the survivor sample, though this limitation cannot be fully resolved without prospective designs enrolling participants in childhood or early adulthood. Within the follow‐up period, death was modeled as a competing outcome in the multinomial framework, mitigating the within‐study competing risk of mortality on dementia incidence. Regarding selection bias from retrospective recall, childhood speech impairment was assessed via a single self‐report item, and systematic differences in recall propensity cannot be fully excluded. Restricting the analytical sample to cognitively intact respondents at baseline reduces the potential for dementia‐related memory deterioration to affect measures of childhood conditions. To the extent that any residual misclassification is non‐differential with respect to prospectively ascertained dementia status, effect estimates remain conservative. Taken together, both sources of bias operate toward the null, suggesting the reported associations are likely underestimates of the true relationship between childhood speech impairment and late‐life dementia risk.

This study has several limitations. First, the measure of speech impairment was not based on clinical diagnosis and may yield biases based on health literacy or memory. Second, although several studies have demonstrated that HRS cognitive tests have high precision with diagnosis reports (Gianattasio et al. 2019, Langa et al. 2020), we must acknowledge the possibility of misclassification. Moreover, speech impairment measure in this study captures speech and language issues in childhood and may ignore the severity or progressive nature of sensory impairments. Future research should explore multiple aspects and specific types of speech impairment as well as other pathways to understand older adults’ cognitive health in later life. As an observational longitudinal cohort study, the present design does not permit causal inference. The associations reported reflect adjusted statistical relationships between childhood speech impairment and dementia incidence and should not be interpreted as evidence of a direct causal effect. Unmeasured confounding and structural limitations inherent to retrospective exposure measures preclude definitive causal attribution.

A further limitation concerns the possibility of shared etiology between childhood speech impairment and late‐life dementia. Both conditions may partly reflect common neurodevelopmental vulnerabilities, including genetic predisposition to neurodegeneration or perinatal insults. Although we adjusted for childhood family SES, childhood self‐rated health, and midlife health conditions, parental cognitive or dementia history was unavailable in the HRS. Residual confounding by shared hereditary factors cannot be excluded, and findings should be interpreted as a robust statistical association rather than a causal effect. Future studies could leverage HRS genetic and biomarker data, including APOE genotype, polygenic risk scores, and blood‐based neuropathological markers, to formally test whether the association persists after accounting for genetic predisposition to neurodegeneration.

To our knowledge, this is the first study to use a nationally representative dataset to examine the relationship between early life speech impairment and the onset of dementia. Findings of the current study expands the evidence base for dementia prevention by identifying early life speech impairment as a potential risk factor, complementing established factors such as depression, midlife hypertension, physical inactivity, and diabetes (Deckers et al. 2015). Understanding this connection allows for earlier identification of at‐risk individuals, which serves as the first step in slowing or mitigating the progression of cognitive decline. In addition, our findings have important implications for early intervention services. Childhood speech impairment may contribute to long‐term socioeconomic disadvantage, exacerbating financial barriers to healthcare access and creating a cycle of disadvantage that reinforces the social gradient in health outcomes from an early age (Law et al. 2013). Early interventions that are frequent, intense, and systematic—promoting engagement and attention within a supportive and positive environment while addressing normal language needs—have been shown to facilitate positive outcomes for children with speech impairment and help avert or reduce substantial and ongoing costs to both individuals and society (Nippold 2012). Childhood speech impairment may serve as an observable early‐life marker of elevated dementia risk in later life, independent of socioeconomic background and childhood health status. Integrating speech and language screening into routine pediatric and early educational assessments could support identification of individuals who may benefit from monitoring of cognitive trajectories and modifiable risk factors across the life course.

## 5 Conclusion

In conclusion, using nationally representative data from HRS, this study is among the first to examine the implications of experiencing speech impairment in childhood for cognitive functioning among U.S. older adults. Findings demonstrated that older adults have a higher risk of dementia if they had experienced speech impairment in childhood. Stressors at midlife including cardiometabolic and depressive symptoms partially explained why early life speech impairment has a long‐lasting implication on later life cognitive health. Increased screening and intervention programs are needed for children and adolescents who have speech or language problems. For those who are living with speech impairments, we should pay attention to their cardiovascular and mental health conditions in order to reduce the risk of dementia.

## Funding

Authors declare that no funding was received for this study. No sponsor is involved in the study design and manuscript preparation.

## Ethics Statement

The data has full ethical approval from UM Health Sciences/Behavioral Sciences IRB Protocol: HUM00061128.

## Conflicts of Interest

Authors have no competing interests to declare.

## Data Availability

The data that support the findings of this study are publicly available as the Health and Retirement Study (HRS) at https://hrs.isr.umich.edu/, produced and distributed by the University of Michigan with funding from the National Institute on Aging (grant number NIA U01AG009740). Ann Arbor, MI.
